# Understanding Parents’ Roles in Children’s Learning and Engagement in Informal Science Learning Sites

**DOI:** 10.3389/fpsyg.2021.635839

**Published:** 2021-03-31

**Authors:** Angelina Joy, Fidelia Law, Luke McGuire, Channing Mathews, Adam Hartstone-Rose, Mark Winterbottom, Adam Rutland, Grace E. Fields, Kelly Lynn Mulvey

**Affiliations:** ^1^Department of Psychology, North Carolina State University, Raleigh, NC, United States; ^2^Department of Psychology, University of Exeter, Exeter, United Kingdom; ^3^Department of Biological Sciences, North Carolina State University, Raleigh, NC, United States; ^4^Department of Science Education, University of Cambridge, Cambridge, United Kingdom; ^5^Department of Education, Liberty University, Lynchburg, VA, United States

**Keywords:** informal science learning, science education, parents, family visits, children

## Abstract

Informal science learning sites (ISLS) create opportunities for children to learn about science outside of the classroom. This study analyzed children’s learning behaviors in ISLS using video recordings of family visits to a zoo, children’s museum, or aquarium. Furthermore, parent behaviors, features of the exhibits and the presence of an educator were also examined in relation to children’s behaviors. Participants included 63 children (60.3% female) and 44 parents in 31 family groups. Results showed that parents’ science questions and explanations were positively related to children observing the exhibit. Parents’ science explanations were also negatively related to children’s science explanations. Furthermore, children were more likely to provide science explanations when the exhibit was not interactive. Lastly there were no differences in children’s behaviors based on whether an educator was present at the exhibit. This study provides further evidence that children’s interactions with others and their environment are important for children’s learning behaviors.

## Introduction

Informal science learning sites (ISLS), such as museums and zoos, are central resources where both children and adults can learn about science (National Research Council, 2009). Findings suggest that optional science experiences outside of formal school environments are associated with science attitudes and knowledge (Liu and Schunn, 2018). Further, recent research with ISLS visitors highlights that children and adults perceived that they learned more when interacting with an educator, especially a youth educator (e.g., a teen docent), rather than the exhibit alone (Mulvey et al., 2020). Additionally, parent-child interactions in informal science learning environments can create important opportunities for learning (Benjamin et al., 2010; Callanan et al., 2017, 2020). Furthermore, according to Social Cognitive Theory the environment and the behaviors of others can play an influential role in children’s learning (Bandura, 1986). Therefore, the aim of the current study was to analyze how parents’ behaviors foster children’s opportunities for learning in different informal learning sites and examine how the interactive features of the exhibits, as well as whether an educator is present at the exhibit, are related to children’s learning behaviors.

### Theoretical Framework

This work was informed by Social Cognitive Theory, which describes how behaviors, environments, and personal characteristics influence the learning process (Bandura, 1986). Prior research has used Social Cognitive Theory to study children’s learning in more formal settings, for instance in schools (Burns et al., 2018). This research has focused on students’ academic achievement, and career orientation, with attention to factors such as social support from peers and parents and teachers and personal factors such as self-efficacy and perceived control (Nugent et al., 2015; Burns et al., 2018). However, Social Cognitive Theory can also be used to understand children’s behaviors in informal learning sites such as science museums. In these settings the environment can influence children’s behaviors. For example, many ISLS have specific environments structured to foster learning, for example these sites include exhibits where children can engage in learning behaviors such as physically interacting with and manipulating structures (Sandifer, 2003; Shaby et al., 2017). Additionally, these exhibits often have educators present who will help children understand how to use these interactive exhibits and also encourage science conversations. Furthermore, social interactions between children and parents can also be influential for children’s behaviors. At these sites, parents’ behaviors can be especially important as they can help children learn and acquire new skills. For example, when parents used questions to guide their children’s learning, children were more likely to engage in scientific processes such as predicting and evaluating (Vandermaas-Peeler et al., 2018). Therefore, Social Cognitive Theory was applied to the current study to evaluate relationships between behaviors (parents’ questions and explanations), the environment (exhibit features and presence of an educator) and children’s learning in ISLS.

### Children’s Learning Behaviors in ISLS

Traditionally, learning has been measured as an accumulation of new information (Hooper-Greenhill, 2003). However, in line with Social Cognitive Theory, children’s behaviors are also important indicators of learning (Bandura, 1986). In ISLS, behaviors such as observing the exhibit, engaging with the exhibit, asking questions, and giving explanations can demonstrate that children are learning.

Children can learn in ISLS by exploring an exhibit in many different ways. Barriault and Pearson (2010) considered behaviors such as observing the exhibit or physically interacting with the exhibit to be “initiation behaviors”–the first steps in learning. There are many opportunities for children to interact with the ISLS through physical manipulation of elements and/or participatory activities. Interactive exhibits in ISLS that place emphasis on hands on learning, have been shown to be very effective in promoting learning in children (Andre et al., 2017). Furthermore, hands-on exploration can also involve using prior knowledge to make connections or understand causal relationships (Legare, 2014). Therefore, children can engage with an exhibit through physical interaction or making connections to other knowledge. These experience-rich environments, that encourage children’s exploration, allow for the use of processes such as evaluation and comprehension, which have been associated with children’s cognitive development (French, 2004).

As indicated above, children’s social interactions in ISLS are essential for their learning. The conversations that children have with educators or their parents may allow them to think more deeply about the information in the exhibits. When children ask questions or give explanations, they explore and make meaning of new information and ideas (Barriault and Pearson, 2010). In these types of interactions children advance their understanding of the concepts encountered in the ISLS, which can promote their science engagement and learning. In the current study, we aim to examine how learning behaviors exhibited by children visiting ISLS with their family are associated with environmental factors, parental behaviors, as well as the prescence of an educator. Although some prior research has examined environmental factors, such as the exhibit features (Barriault and Pearson, 2010; Shaby et al., 2017), explored children’s learning with attention to the role of the parents (Benjamin et al., 2010; Callanan et al., 2020), and other research has examined children’s interactions with educators in ISLS (Shaby et al., 2019), scant research has attended to both caregivers and educators as well as environmental factors in concert.

### Environmental Factors Related to Children’s Learning

In addition to considering the role of parents and educators, it may also be important to focus on environmental features when examining children’s learning in ISLS. Although we often think of learning environments with attention to formal classroom spaces, informal spaces are rich environments that can create opportunities for learning. When families visit ISLS, they are exposed to a range of ways to engage with novel environments in exhibits, providing ample opportunities for science learning to occur. Although many visitors report that the primary reason why they visit ISLS is for entertainment (Tofield et al., 2003), ISLS also provide opportunities for science learning (National Research Council, 2009; Shouse et al., 2010). Research has focused on the academic- and science-related outcomes of visiting ISLS, including attention to learning during school group (Tal and Morag, 2007; Shaby and Vedder-Weiss, 2019; Shaby et al., 2019) and family visits (Benjamin et al., 2010; Haden, 2010; Callanan et al., 2017, 2020; Pattison et al., 2018).

Students can gain critical science skills and have opportunities for practical application when they visit ISLS (Bell et al., 2009). Experiences in ISLS also provide other academic benefits including increased academic aspirations, increased interest in math and science, and feelings of competency in science (Lin and Schunn, 2016; Goff et al., 2019). Furthermore, there are often multiple interactive features in ISLS, and this interactivity is associated with longer visitor engagement (Sandifer, 2003; Shaby et al., 2017). Interactive exhibits may have features which children can physically interact with, whereas non-interactive exhibits facilitate observing behaviors. A study that analyzed parent-child dyads at an ISLS found that parents and children spent more time at exhibits that were interactive compared to non-interactive exhibits (Szechter and Carey, 2009). Therefore, interactive exhibits are critical for visitor engagement. Not only are the interactions with the exhibit influential, but the social interactions that occur in ISLS are also key factors associated with science learning in children. In the present study, we examined environmental factors across a range of different types of ISLS and exhibits, including exhibits in a zoo, a children’s museum and an aquarium. Thus, we further extend prior work, which has often examined children’s learning within one type of setting. Our aim was, in part, to document what environmental factors are associated with children’s learning across different types of settings and exhibits. This is a critical new direction for research as it can help to document best practices for museum design and exhibit development that reach across the silos that are often formed within particular types of learning settings.

### What Role Do Educators Play in Children’s Learning?

Educators can aid visitor learning by providing explanations, asking questions, and instructing visitors on how to use the exhibit. For example, in one study, when educators gave tips on how to build a structure in a building activity, children used more science-related talk when recalling their museum visit compared to children who did not receive any tips (Haden et al., 2014). Additionally, Mulvey et al. (2020) found that child and adult visitors felt that they learned more from their experiences in ISLS after interacting with an educator rather than just the exhibit. Visitors in that study also reported greater interest in the topic after interacting with a youth educator compared to an adult educator. Educators also encourage learning by emotionally engaging with guests to increase interest in the exhibits (Shaby et al., 2019). Furthermore, another study found that, compared to visitors in a greeting condition, in which educators simply greeted guests when they approached an interactive math exhibit, visitors who interacted in more substantial ways with educators spent more time at the exhibit, felt more satisfied with their experience, and had a better understanding of the content, including mathematical reasoning in particular (Pattison et al., 2018). However, this study also found that, when educators were present, parents and children were less likely to communicate with each other. This suggests that in the presence of educators, instead of talking to each other, parents and children may direct questions and comments toward the educators which may hinder key parent-child interactions that are important for children’s learning. Therefore, it is important to explore both educators’ and parents’ behaviors in ISLS together.

### How Are Parents’ Behaviors Related to Their Children’s Learning?

Parent-child conversations can promote children’s learning (Crowley et al., 2001; Fender and Crowley, 2007). For example, in one study, parents’ use of explanatory conversations, such as providing scientific explanations or asking questions, was positively related to their children’s use of explanatory conversations at an evolution exhibit (Tare et al., 2011). These types of conversations can help keep children engaged and promote children’s scientific dialog. However, parents’ explanations and questions can elicit different behaviors for their children.

In a study exploring children’s and parents’ conversations in a museum, parents’ requests for explanations from their children were positively related to children’s engaged talk (requests and explanations), but parents’ explanations were negatively related to children’s engaged talk (Callanan et al., 2017). Furthermore, when parents were instructed to provide either scientific questions or statements, children whose parents asked more scientific questions responded more to their parents compared to children whose parents gave scientific statements (Chandler-Campbell et al., 2020). Additionally, when parents asked scientific questions, their children were more likely to answer with scientific responses. However, parents’ explanations have been shown to often be incomplete or incorrect (Snow and Kurland, 1996; Crowley et al., 2001) parents may not know enough about certain concepts to accurately explain them, which can create more confusion and misunderstanding for their children.

When parents ask their children questions, rather than just providing answers, they are more likely to create meaningful conversations (Callanan et al., 2017). Asking questions, especially open-ended questions, can help parents and other educators to understand what children know while facilitating children’s learning of new information (Haden, 2010). Although parents’ explanations may not always promote learning, children’s use of explanations can (Booth et al., 2020). Research has also shown that parents’ invitations to their children to provide their own causal explanations were related to their children’s scientific literacy (Booth et al., 2020). Thus, creating opportunities that allow children to think critically and engage with the material promotes children’s learning (Haden, 2010). The present study extends previous work on the effectiveness of parents’ explanations and requests on their children’s own scientific talk.

## Current Study

Informed by Social Cognitive Theory, the present study used observational video-based data to analyze how the environment and the presence of educators at ISLS as well as parents’ behaviors are related to children’s learning behaviors. The children’s behaviors we evaluated were children’s observations of the exhibit, engagement with the exhibit, requests for science information, and use of science explanations. Thus, we evaluated how parent-child conversations, the presence of an educator, the length of time of the visit, and how interactive exhibits in ISLS relate to children’s conversations and behaviors.

### Hypotheses

(1) Based on findings from Mulvey et al. (2020), we expected that the presence of an educator would be positively related to all children outcome variables (children observing the exhibit, children engaging with the exhibit, children’s requests for science information, and children’s science explanations).
(2) In a non-interactive exhibit in which parents use more science explanations we expected that children would be more likely to observe the exhibit (Callanan et al., 2017).
(3) Given prior research that demonstrates how effective interactive exhibits are for children’s learning (Andre et al., 2017), we expected that if the exhibit is interactive, children would be more likely to engage with the exhibit.
(4) As findings suggest that when parents ask questions, children are more likely to engage in explanatory conversations (Tare et al., 2011), we expected that parents’ requests for science information would be associated with more requests for science information and explanations from children.
(5) Moreover, in line with findings from Callanan et al. (2017), we expected that parents’ science explanations would be associated with fewer requests for science information and explanations from children.

## Materials and Methods

### Participants

In this study we analyzed 31 video recordings of the interactions between children, their parents, educators and the exhibit itself. Thirteen of the videos had an educator present at the exhibit. Participants included 31 families of 63 children (∼60.3% female) and 44 parents (∼76.9% female). Twenty-one family groups included more than one child and the average number of children per family group was 2.03. We were unable to directly request demographic data from families and thus demographic information including age, gender, and ethnicity were coded based on inferences made by the research assistants coding the videos. We estimated that roughly 43% of youth visitors were in early childhood (3–8 years), 44% percent were in middle childhood (9–13 years), and 13% were 14 or older. We also estimated that roughly 60% of families were White. All participants spoke English in the videos.

### Procedure

This research was approved as Exempt by the Institutional Review Board at the University of South Carolina with an Inter-Institutional Agreement by North Carolina State University. Participants were recruited from three different ISLS: a zoo, an aquarium, and a children’s museum (see Table 1 for descriptions of exhibits) in the Southeastern United States. Signs were posted about the research project at the entrance to the exhibits and participants were invited to participate by a research assistant and provided with a notification letter about the study. If the family agreed to participate, they were asked to wear a microphone headset and were video recorded while visiting selected exhibits. Educators also wore a lapel microphone. Video cameras were placed in three locations at each exhibit to ensure that the full family visit was recorded.

**TABLE 1:** Exhibit descriptions.

Site	Number of Videos at Site	Exhibits	Type of Exhibit	Description
Aquarium	11	Reptile Exhibit	Non-interactive	Visitors were able to view animals such as a Komodo dragon and a Tomistoma. Exhibit signage provided information on the habitats and ecology of the animals. Educators were at times present to provide additional information about the species and their ecology.
Children’s Museum	8	Flight Exhibit	Interactive	Visitors could make a paper airplane and could test out their airplanes by throwing them through hoops hung from the ceiling. Educators would help visitors build their paper airplanes and discuss principles of flight. Visitors could also use a flight simulator to pretend to fly an airplane.
Zoo	12	Gorilla Exhibit	Interactive	Visitors could view the gorillas in an outdoor exhibit, use interactive maps and other displays to learn about the specific gorillas at the zoo as well as the dangers facing wild gorillas. Educators at this exhibit taught using “biofacts” such as a gorilla skull and share information, also available on exhibit signage, about the places that gorillas live, the food they eat, and other information about gorillas.
		Sea Lion and Seal Exhibit	Interactive	Visitors could observe the sea lions and seal on two levels, through large glass panels. The exhibit included an artistic display of trash found in the ocean that visitors could look at and touch. This display was used to demonstrate the pollution in the ocean. Educators also provide interpretation, sharing similar information about sea lion and seal ecology as is found on exhibit signage.

### Coding and Transcription

All data were transcribed by trained research assistants and videos were coded in Atlas.ti (ATLAS.ti Scientific Software Development GmbH) using a coding system developed based on those used in two prior studies (Barriault and Pearson, 2010; Callanan et al., 2017). Each interaction (see Table 2 for descriptions of measures coded) for each person was coded once in 30s intervals. For example, if a child asked two science requests in the first 30s of the video, the code of “science request” for that child was used once for that interval. Scores for each interaction type were determined by summing the instances of the behavior for parents and children during the time spent at the exhibit. Each video was coded by two research assistants, and the interrater reliability (as calculated in Atlas.ti) was 82.54%. The duration of each video ranged from 30 s to 7 min.

**TABLE 2:** Descriptions of measures.

Measure	Definitions
Children Observing the Exhibit	Refers to when someone is looking at the exhibit without interacting or talking, or looking at others engaging with the exhibit.
Children Engaging with the Exhibit	Refers to when someone is physically using the devices or educational materials at an exhibit or when someone is providing additional information that connects to prior knowledge. Example: children could make a paper airplane in the flight exhibit. Example: “I read about Gorillas in a book, they live there.”
Children and Parents’ Requests for Science Information	Defined as asking for an explanation relevant to the science exhibit or requesting evidence for a claim/conclusion. Example: (Flight exhibit) “What is knots? Is it like a measurement?”
Children and Parents’ Science Explanations	Defined as making an explanation relevant to the science exhibit or using evidence to draw a conclusion. Example: (Gorilla exhibit) “That is a termite mound. The gorillas will use their teeth to make tools which they will stick inside of the termite mound.”
Interactive Exhibit	Interactive exhibits featured objects that visitors could touch or activities that visitors could participate in, whereas non-interactive exhibits could only be observed.
Duration	The total length of time in seconds that a child spent at the exhibit.
Educator Condition	Videos were coded for whether an adult, youth, or no educator was present during the children’s visit to the exhibits.

### Data Analysis Plan

Since participants visited different ISLS sites, and as children were nested in family groups, multilevel modeling was used to account for the nesting of data. Furthermore, multi-level modeling approaches are robust with as few as 10 groups in level 2, especially if restricted maximum likelihood and the Satterthwaite approximation are used (Huang, 2018). Multilevel models were fit using the MIXED command in SPSS with restricted maximum likelihood and the Satterthwaite approximation in order to assess children’s science explanations, requests for science information, and whether they were engaging with the exhibit, and observing the exhibit. Educator condition (no educator, youth educator, adult educator), interactive exhibit (yes, no), parents’ science explanations, parents’ requests for science information, and duration spent at the exhibit were used as fixed effects. The site ID and family ID were used as random effects. The equations for the multilevel models were as follows:

$$\gamma_{i\,j\,k}=\gamma_{00}+\gamma_{01}\,P\,a\,r\,e\,n\,t\,s\,S\,c\,i\,R\,e\,q\,u\,e\,s\,t_{i\,j}+\gamma_{02}\,P\,a\,r\,e\,n\,t\,s\,S\,c\,iE\,x\,p\,l\,a\,n\,a\,t\,i\,o\,n_{i\,j}+\gamma_{000}\,D\,u\,r\,a\,t\,i\,o\,n_{j}+\gamma_{002}\,I\,n\,t\,e\,r\,a\,c\,t\,i\,v\,e\,E\,x\,h\,i\,b\,i\,t_{j}+\gamma_{003}\,E\,d\,u\,c\,a\,t\,o\,r\,C\,o\,n\,d\,i\,t\,i\,o\,n_{j}+e_{0\,i\,j\,k}+u_{0\,j\,k}+u_{00}$$

The outcome for the *i*th visitor in the *j*th site and *k*th family group is modeled as main effect of parents’ science requests (γ_01_), parents’ science explanations (γ_02_), duration in seconds spent at the exhibit (γ_000_), interactive exhibit (γ_002_), and educator exhibit (γ_003_), with γ_00_ as the overall mean and u_0*jk*_ and u_00_ as the family group and site residuals and *e_0ijk_* as the individual residuals. This general equation was tested for each of the dependent variables (Table 3).

**TABLE 3:** Intra-class correlation coefficients accounting for family group and site level variance in key dependent variables.

Dependent Variable	Family Group ICC	Site ICC
Children Observing the Exhibit	0.42	0.05
Children Engaging with the Exhibit	0.89	0.61
Children’s Requests for Science Information	0.16	0.06
Children’s Science Explanations	0.06	0.13

## Results

### Descriptives

Parents gave more science explanations when an educator was not present, *t*(61) = 4.73, *p* < 0.001, and parents made less requests for science information when an educator was not present, *t*(61) = −2.54, *p* < 0.001. In fact, parents only made requests for science information when an educator was present. Children also gave more science explanations when an educator was not present, *t*(61) = 1.66, *p* = 0.001. Although not significant, children observed the exhibit more and made more requests for science information when an educator was present, however they engaged with the exhibit more when an educator was not present (Table 4). Additionally, children observing the exhibit was negatively associated with their exhibit engagement and positively associated with parents’ requests for science information (Table 5).

**TABLE 4:** Means and ranges for children’s and parents’ behaviors when an educator was present or not.

	Children Observing the Exhibit	Children Engaging with the Exhibit	Children’s Requests for Science Information	Children’s Science Explanations	Parents’ Requests for Science Information	Parents’ Science Explanations	Duration
Educator Present	1.09	1.40	0.24	0.04	0.78	0.11	148.80
No Educator	0.61	1.44	0.17	0.22	0.00	0.78	81.6
Range	0–5	0–8	0–2	0–2	0–5	0–2	30–420

*Means represent the sum of the number of instances across the exhibit visit of the particular behavior. Duration is measured in seconds.*

**TABLE 5:** Correlations.

Variable	1	2	3	4	5	6	7	8	9
Children Observing the Exhibit	–								
Children Engaging with the Exhibit	−0.24*	–							
Children’s Requests for Science Information	−0.05	0.11	–						
Children’s Science Explanations	−0.06	−0.01	−0.10	–					
Parents’ Requests for Science Information	0.55**	−0.13	0.01	−0.08	–				
Parents’ Science Explanations	0.24	0.16	0.09	−0.13	−0.01	–			
Interactive Exhibit	−0.13	0.04	0.16	−0.26*	−0.22	−0.33**	–		
Duration	0.34**	0.58**	0.01	−0.13	0.36**	0.23	−0.13	–	
Educator Condition	0.03	−0.10	0.00	−0.16	0.17	−0.51**	0.09	0.06	–

*p < 0.05, **p < 0.01.

### Children Observing the Exhibit

Parents’ science explanations (*b* = 0.74, *t* = 2.19, *p* = 0.04) and requests for science information (*b* = 0.54, *t* = 3.67, *p* = 0.001) were related to children observing the exhibit. When children were observing the exhibit, parents were more likely to request science information and give more science explanations. No other variables were significantly related to children observing the exhibit (Table 6).

**TABLE 6:** Unstandardized coefficients (and standard errors) of multilevel models of children’s behaviors.

Effect	Parameter	Children Observing the Exhibit	Children Engaging with the Exhibit	Children’s Requests for Science Information	Children’s Science Explanations
Intercept	γ00	0.47(0.37)	0.78(0.70)	0.38(0.20)	−0.05(0.11)
Parents’ Requests for Science Information	γ01	0.54**(0.15)	−0.47(0.27)	0.003(0.08)	−0.03(0.05)
Parents’ Science Explanations	γ02	0.74*(0.34)	−0.67(0.62)	0.30(0.19)	−0.29**(0.11)
Duration	γ001	0.003(0.06)	0.44**(0.12)	−0.02(0.03)	0.001(0.02)
Interactive Exhibit	γ002	−1.67(0.37)	−1.56*(0.71)	−0.26(0.21)	0.31**(0.01)
Educator Condition	γ003	0.20(0.42)	−0.66(0.78)	0.00(0.23)	0.11(0.12)
Random Effects					
Family ID		0.88^**,*⁣**^(0.20)	0.47^**,*⁣**^(0.12)	0.24^**,*⁣**^(0.06)	0.12^**,*⁣**^(0.02)
Site ID		0.24(0.19)	2.26**(0.71)	0.08(0.07)	0.00(0.00)

*p < 0.05, **p < 0.01, *** *p* < 0.001.

### Children Engaging With the Exhibit

Interactive exhibit (*b* = −1.56, *t* = −2.18, *p* = 0.04) and duration (*b* = 0.44, *t* = 3.72, *p* = 0.001) were significantly related to children engaging with the exhibit. If the exhibit was not interactive, children were less likely to engage with the exhibit and the longer children spent at the exhibit the more likely they were to engage with the exhibit. No other variables were significantly related to children engaging with the exhibit (Table 6).

### Children’s Requests for Science Information

Children’s requests for science information were not related to any of the variables (Table 6).

### Children’s Science Explanation

There was a significant effect of interactive exhibit (*b* = 0.31, *t* = 2.71, *p* = 0.01) on children’s science explanation. If the exhibit was not interactive, children were more likely to give science explanations. There was also a significant negative effect of parents’ science explanation (*b* = −0.29, *t* = −2.76, *p* = 0.01) on children’s science explanations. This suggests that the more parents gave science explanations the less children gave science explanations. No other variables were significantly related to children’s science explanation (Table 6).

## Discussion

This study evaluated the interactions between parents and children in ISLS, by examining how parents’ scientific questions and statements, as well as the aspects of the exhibit such as the presence of educators and interactive materials, are related to children’s learning behaviors. Importantly, this study examined children’s learning across different types of exhibits and sites, documenting common patterns of learning across an aquarium, a zoo and a children’s museum. Consistent with Social Cognitive Theory (Bandura, 1986), we found that behaviors and environment are related to learning: parents’ requests for science information and interactive exhibits may be important factors associated with learning behaviors in children. Results showed that when parents asked more science questions, children were more likely to observe the exhibit. Parents’ frequency of science explanations was also positively related to children observing the exhibit, but they were negatively related to children’s science explanations. None of the variables were related to children’s requests for science information and duration was only related to children engaging with the exhibit. Furthermore, if the exhibit was not interactive, children were more likely to provide science explanations and were less likely to engage with the exhibit. We did not find differences in children’s behaviors based on whether an educator was present at the exhibit. This was somewhat surprising given previous studies that reported feelings of learning more at an exhibit when an educator was present–especially a youth educator (Mulvey et al., 2020).

### Interactive Exhibits

As expected based on Social Cognitive Theory, we found that environment plays an important role in the types of learning behaviors children display. Children were more engaged, through physical interaction or providing additional information, with the exhibit when the exhibit included interactive elements. This is consistent with findings that show that when ISLS allow for exploration, visitors are more likely to be engaged through their interactions with the exhibits (Sandifer, 2003; Shaby et al., 2017). We also found that the longer children spent at an exhibit, the more likely they were to engage with the exhibit, which supports previous findings that visitors tend to spend more time at an exhibit that is interactive (Szechter and Carey, 2009). Hands-on exhibits like these, where children get to interact with the exhibits, can help facilitate the first steps of children’s learning (Barriault and Pearson, 2010). However, it is important to note that learning does occur in different ways. When exhibits were not interactive, children were more likely to provide scientific explanations. Thus, it may be that when children encounter non-interactive exhibits, they spend more time considering scientific concepts or generating explanations related to the exhibit content.

### The Presence of Educators

There were no significant relationships between the presence of educators and children’s behaviors. Although not significantly different, descriptive data showed a trend of children asking more science questions when an educator was present. For example, the following illustrates a conversation between a child and an adult educator at the gorilla exhibit at the zoo:

Child: “Do they [gorillas and chimpanzees] live in two places?”

Adult Educator: “Yes the Lowland Gorillas live here [pointing to map], and the chimpanzees live in central Africa, but both of them live in Western Africa.”

Therefore, future research may more carefully explore educator behaviors that encourage children’s requests for science information. As prior research has documented the relationship between educators and visitors’ understanding of science concepts and use of science related dialog (Haden et al., 2014; Pattison et al., 2018), it is important that future work continue to explore what types of learning educators foster. We also did not find differences based on whether an educator was present or not for children’s observation or any other outcomes. This may be because educators vary in the ways that they engage with visitors. For example, it may be interesting for future research to examine differences in educators’ use of science requests and explanations.

### How Parents’ Behaviors Are Related to Their Children’s Learning

Also in alignment with Social Cognitive Theory, we found that parents’ behaviors, specifically their use of science explanations and requests for science information were related to children’s behaviors. Parents’ science explanations and requests for science information were positively related to children observing the exhibit. Instances where the child observes animals while parents explain or ask questions were common, since two of our sites were a zoo and an aquarium. Observing animals is a crucial part of these exhibits; thus, this behavior, in this context, may provide rich opportunities for learning. Previous research has shown that when visitors observed scientists conducting research with animals at an exhibit, they reported greater perceived learning (Waller et al., 2012). Through observing exhibits like these, children are able to learn about the animals’ needs, their environments, and research and conservation efforts to protect the species (Tofield et al., 2003).

Although we were unable to quantitatively analyze the data to indicate the directionality of the behaviors between parents and children, this example demonstrates that parents’ behaviors would often promote their children’s behaviors. The following interaction from the aquarium shows a parent’s explanation of science information preceding a child observing the exhibit.

A parent approaches the Komodo dragon exhibit and gives a short description of the animal to their child: “That’s a Komodo dragon. They like to eat dead animals.” The child then approaches the exhibit and observes the animal.

Therefore, observations are important behaviors that allow for the opportunity to learn new information. Our findings reveal the important role of parents while children are observing exhibits–the more parents asked questions and provided science explanations, the more children observed the exhibit. This extends previous research by demonstrating that parents’ science explanations may offer some benefits by encouraging children to engage with exhibits through observation.

We also found that children were less likely to give science explanations when their parents gave science explanations. This finding supports previous research that showed that parents’ science explanations were negatively related to children’s requests and explanations (Callanan et al., 2017). Our finding suggests that for children to be more engaged with the exhibit and express their own knowledge, parents should consider offering fewer explanations and instead let their children lead the exploration more directly. This is demonstrated by the following interaction at the aquarium:

As a child and parent approach the Komodo dragon exhibit, the parent does not immediately offer information about the exhibit. Instead, the child explains while the parent listens: “It’s a giant lizard. They’re really fast, did you know that? Look at the bottom of its neck, you can see it breathing. It’s shedding its skin.”

Interactions like this one allow the child to guide the discussion and display their own knowledge of the exhibit, which can create engaged conversations between parents and children.

Although there was not a significant effect of parents’ requests for science information on children’s science explanations, prior research has documented that asking children questions was related to more scientific and engaged talk from children (Callanan et al., 2017; Chandler-Campbell et al., 2020). However, previous research has also shown that many parents may not know what to ask their children or how to explain certain concepts (Snow and Kurland, 1996; Crowley et al., 2001), which is demonstrated in the example below from the flight exhibit at the children’s museum in which the child is using a flight simulator to fly a plane:

Child: “What is knots? Is Knots like a measurement?”

Parent: “It’s a measurement, for speed, I guess…. or distance.”

In this example the child asked their parent a science related question pertaining to the activity they are engaged in. The parent gives wavering science explanations–one of which is incorrect. This example demonstrates how parents may try to explain concepts but may not always have high perceptions of their own competence in, or foundational knowledge of, these domains. Therefore, providing parents with information regarding the exhibits may be helpful for these conversations.

Studies have shown that providing parents with information or prompts to guide their conversations with their children helps create parent-child conversations (Harris and Winterbottom, 2018). For example, when educators suggested that parents ask more “What?, Why?, Where?, and How?” parents asked twice as many questions to their children, compared to parents who did not receive any conversation instructions (Haden et al., 2014). Thus, instructions or suggestions that provide parents with examples of questions to ask could be very effective in creating conversations between parents and children.

## Limitations and Future Directions

Although our findings provide insight into children and parents’ behaviors in ISLS, we must acknowledge the limitations of our study. This study focused on demonstrating the benefits of spending more time at exhibits, however future research should continue to explore duration as there may be a time point for how long families should spend at an exhibit for optimal learning. Additionally, we were unable to ask families to directly report participant demographics, and thus, were unable to confidently analyze findings based on these. However, it was estimated that the majority of participants were White families, therefore more work is needed that includes members from diverse groups. Further, prior research demonstrates that ethnic minority families often report that ISLS are not “for them” (Dawson, 2014). It would be important for future research to examine differences in parent-child interactions for families of different ethnic backgrounds, as this may provide additional insight into why ethnic minority families feel unwelcome in these sites. Finally, Mulvey et al. (2020) found that visitors felt they learned more when interacting with an educator rather than with just the exhibit. Although we examined the presence and absence of an educator; future research might more carefully examine the specific educator behaviors that encourage children’s learning opportunities. For example, based on our means both children and parents used more requests for science information when an educator was present. Therefore, future research could try to code for the types of educator behaviors that may elicit these responses from visitors.

## Conclusion

This study provides support for Social Cognitive Theory by demonstrating that parents’ behaviors and environment are important factors related to children’s behaviors. It also further expands our understanding of parent-child interactions in ISLS by showing that parents’ science explanations are both positively and negatively associated with children’s learning behaviors. If parents’ goals are to encourage their children’s learning through observations, then providing science explanations would be helpful. However, parents should consider offering fewer explanations in order to encourage children to ask questions or explain concepts. Furthermore, the findings from this study can be used to shape exhibits in ISLS. Our results revealed that children were more likely to provide science explanations when an exhibit was non-interactive, however they were more likely to physically interact with the exhibit or provide additional information when the exhibit was interactive. Therefore, ISLS should focus on creating spaces that have a balance of interactive and non-interactive components as both have their own benefits for children’s learning behaviors. By promoting the use of interactive exhibits, visitors’ can gain more opportunities for learning and engagement. Additionally, ISLS could provide parents with important information about, or discussion prompts for, the exhibits to help guide their discussions and create more meaningful conversations with their children. In sum, these findings document the ways in which parents and children interact in ISLS and reveal the important role that parents play, even when educators are also present in ISLS.

## Data Availability Statement

The raw data supporting the conclusions of this article will be made available by the authors, without undue reservation.

## Ethics Statement

The studies involving human participants were reviewed and approved by University of South Carolina with institutional agreement from North Carolina State University Institutional Review Boards. Written informed consent from the participants’ legal guardian/next of kin was not required to participate in this study in accordance with the national legislation and the institutional requirements.

## Author Contributions

AJ developed the hypotheses, supervised the coding of the videos, performed the statistical analysis, coordinated and drafted the manuscript. FL participated in the study design and development of the coding system and helped to draft the manuscript. LM, MW, and GF participated in the study design and helped to draft the manuscript. CM helped to draft the manuscript. AH-R and AR led the study design and helped to draft the manuscript. KM led the study design and data analytic approach, and helped to draft the manuscript. All authors read and approved the final manuscript.

## Conflict of Interest

The authors declare that the research was conducted in the absence of any commercial or financial relationships that could be construed as a potential conflict of interest.

## Abbreviations

ISLS: informal science learning sites
STEM: science, technology, engineering and mathematics.

## References

[B1] Andre L. Durksen T. Volman M. L. (2017). Museums as avenues of learning for children: a decade of research. *Learn. Environ. Res.* 20 47–76. 10.1007/s10984-016-9222-9

[B2] Bandura A. (1986). *Social Foundations of Thought and Action: A Social Cognitive Theory.* New Jersey, NJ: Prentice-Hall, Inc.

[B3] Barriault C. Pearson D. (2010). Assessing exhibits for learning in science centers: a practical tool. *Visitor Stud.* 13 90–106. 10.1080/10645571003618824

[B4] Bell P. Lewwnstein B. Shouse A. W. Feder M. A. (2009). *Learning Science in Informal Environments: People, Places, and Pursuits.* Washington, DC: National Academies Press.

[B5] Benjamin N. Haden C. A. Wilkerson E. (2010). Enhancing building, conversation, and learning through caregiver–child interactions in a children’s museum. *Dev. Psychol.* 46:502. 10.1037/a0017822 20210509

[B6] Booth A. E. Shavlik M. Haden C. A. (2020). Parents’ causal talk: links to children’s causal stance and emerging scientific literacy. *Dev. Psychol.* 56 2055–2064. 10.1037/dev0001108 32833470

[B7] Burns E. C. Martin A. J. Collie R. J. (2018). Adaptability, personal best (PB) goals setting, and gains in students’ academic outcomes: a longitudinal examination from a social cognitive perspective. *Contemp. Educ. Psychol.* 53 57–72. 10.1016/j.cedpsych.2018.02.001

[B8] Callanan M. A. Castañeda C. L. Luce M. R. Martin J. L. (2017). Family science talk in museums: predicting children’s engagement from variations in talk and activity. *Child Dev.* 88 1492–1504. 10.1111/cdev.12886 28657198

[B9] Callanan M. A. Legare C. H. Sobel D. M. Jaeger G. J. Letourneau S. McHugh S. R. (2020). Exploration, explanation, and parent–child interaction in museums. *Monogr. Soc. Res. Child Dev.* 85 7–137. 10.1111/mono.12412 32175600 PMC10676013

[B10] Chandler-Campbell I. L. Leech K. A. Corriveau K. H. (2020). Investigating science together: inquiry-based training promotes scientific conversations in parent-child interactions [Original Research]. *Front. Psychol.* 11:1934. 10.3389/fpsyg.2020.01934 32849136 PMC7419620

[B11] Crowley K. Callanan M. A. Jipson J. L. Galco J. Topping K. Shrager J. (2001). Shared scientific thinking in everyday parent–child activity. *Sci. Educ.* 85 712–732. 10.1002/sce.1035

[B12] Dawson E. (2014). ‘Not designed for us’: how science museums and science centers socially exclude low−income, minority ethnic groups. *Sci. Educ.* 98 981–1008. 10.1002/sce.21133 25574059 PMC4280489

[B13] Fender J. G. Crowley K. (2007). How parent explanation changes what children learn from everyday scientific thinking. *J. Appl. Dev. Psychol.* 28 189–210. 10.1016/j.appdev.2007.02.007

[B14] French L. (2004). Science as the center of a coherent, integrated early childhood curriculum. *Early Child. Res. Q.* 19 138–149. 10.1016/j.ecresq.2004.01.004

[B15] Goff E. E. Mulvey K. L. Irvin M. J. Hartstone-Rose A. (2019). The effects of prior informal science and math experiences on undergraduate STEM identity. *Res. Sci. Technol. Educ.* 38 1–17. 10.1080/02635143.2019.1627307

[B16] Haden C. A. (2010). Talking about science in museums. *Child Dev. Perspect.* 4 62–67. 10.1111/j.1750-8606.2009.00119.x

[B17] Haden C. A. Jant E. A. Hoffman P. C. Marcus M. Geddes J. R. Gaskins S. (2014). Supporting family conversations and children’s STEM learning in a children’s museum. *Early Child. Res. Q.* 29 333–344. 10.1016/j.ecresq.2014.04.004

[B18] Harris E. Winterbottom M. (2018). ‘Why do parrots talk?’ co-investigation as a model for promoting family learning through conversation in a natural history gallery. *J. Biol. Educ.* 52 89–100. 10.1080/00219266.2017.1408934

[B19] Hooper-Greenhill E. (2004). Measuring learning outcomes in museums, archives and libraries: The learning impact research project (LIRP). *Int. J. Herit. Stud*. 10 151–174. 10.1080/13527250410001692877

[B20] Huang F. L. (2018). Multilevel modeling myths. *School Psychol. Q.* 33:492. 10.1037/spq0000272 30070555

[B21] Legare C. H. (2014). The contributions of explanation and exploration to children’s scientific reasoning. *Child Dev. Perspect.* 8 101–106. 10.1111/cdep.12070

[B22] Lin P.-Y. Schunn C. D. (2016). The dimensions and impact of informal science learning experiences on middle schoolers’ attitudes and abilities in science. *Int. J. Sci. Educ.* 38 2551–2572. 10.1080/09500693.2016.1251631

[B23] Liu A. S. Schunn C. D. (2018). The effects of school-related and home-related optional science experiences on science attitudes and knowledge. *J. Educ. Psychol.* 110 798–810. 10.1037/edu0000251

[B24] Mulvey K. L. McGuire L. Hoffman A. J. Goff E. Rutland A. Winterbottom M. (2020). Interest and learning in informal science learning sites: differences in experiences with different types of educators. *PLoS One* 15:e0236279. 10.1371/journal.pone.0236279 32701956 PMC7377401

[B25] National Research Council (2009). *Learning Science in Informal Environments: People, Places, and Pursuits.* Washington, DC: National Academies Press.

[B26] Nugent G. Barker B. Welch G. Grandgenett N. Wu C. Nelson C. (2015). A model of factors contributing to STEM learning and career orientation. *Int. J. Sci. Educ.* 37 1067–1088. 10.1080/09500693.2015.1017863

[B27] Pattison S. A. Rubin A. Benne M. Gontan I. Andanen E. Shagott T. (2018). The impact of facilitation by museum educators on family learning at interactive math exhibits: a quasi-experimental study. *Visitor Stud.* 21 4–30. 10.1080/10645578.2018.1503879

[B28] Sandifer C. (2003). Technological novelty and open-endedness: two characteristics of interactive exhibits that contribute to the holding of visitor attention in a science museum. *J. Res. Sci. Teach.* 40 121–137. 10.1002/tea.10068

[B29] Shaby N. Assaraf O. B.-Z. Tal T. (2017). The particular aspects of science museum exhibits that encourage students’ engagement. *J. Sci. Educ. Technol.* 26 253–268. 10.1007/s10956-016-9676-7

[B30] Shaby N. Ben-Zvi Assaraf O. Tal T. (2019). An examination of the interactions between museum educators and students on a school visit to science museum. *J. Res. Sci. Teach.* 56 211–239. 10.1002/tea.21476

[B31] Shaby N. Vedder-Weiss D. (2019). Science identity trajectories throughout school visits to a science museum. *J. Res. Sci. Teach*. 57 733–764. 10.1002/tea.21608

[B32] Shouse A. Lewenstein B. V. Feder M. Bell P. (2010). Crafting museum experiences in light of research on learning: implications of the national research council’s report on informal science education. *Curator* 53 137–154. 10.1111/j.2151-6952.2010.00015.x

[B33] Snow C. E. Kurland B. (1996). “Sticking to the point: talk about magnets as a context for engaging in scientific discourse,” in *Discourse, Learning, and Schooling*, ed. Hicks D. (Cambridge: Cambridge University Press), 189–220. 10.1017/cbo9780511720390.007

[B34] Szechter L. E. Carey E. J. (2009). Gravitating toward science: parent-child interactions at a gravitational-wave observatory. *Sci. Educ.* 93 846–858. 10.1002/sce.20333

[B35] Tal T. Morag O. (2007). School visits to natural history museums: teaching or enriching? *J. Res. Sci. Teach.* 44 747–769. 10.1002/tea.20184

[B36] Tare M. French J. Frazier B. N. Diamond J. Evans E. M. (2011). Explanatory parent–child conversation predominates at an evolution exhibit. *Sci. Educ.* 95 720–744. 10.1002/sce.20433

[B37] Tofield S. Coll R. K. Vyle B. Bolstad R. (2003). Zoos as a source of free choice learning. *Res. Sci. Technol. Educ.* 21 67–99. 10.1080/02635140308342

[B38] Vandermaas-Peeler M. Westerberg L. Fleishman H. Sands K. Mischka M. (2018). Parental guidance of young children’s mathematics and scientific inquiry in games, cooking, and nature activities. *Int. J. Early Years Educ.* 26 369–386. 10.1080/09669760.2018.1481734

[B39] Waller B. M. Peirce K. Mitchell H. Micheletta J. (2012). Evidence of public engagement with science: visitor learning at a zoo-housed primate research centre. *PLoS One* 7:e44680. 10.1371/journal.pone.0044680 23028580 PMC3441616

